# A positive feedback loop between tryptophan hydroxylase 1 and β-Catenin/ZBP-89 signaling promotes prostate cancer progression

**DOI:** 10.3389/fonc.2022.923307

**Published:** 2022-09-12

**Authors:** Chengguo Ge, Jiusong Yan, Xiaoyu Yuan, Guangyong Xu

**Affiliations:** Department of Urology, Second Affiliated Hospital, Chongqing Medical University, Chongqing, China

**Keywords:** tryptophan hydroxylase 1, 5-hydroxytryptamine, beta-catenin, ZBP-89, prostate cancer

## Abstract

Alterations in tryptophan (Trp) metabolism facilitate the continuous modulation of tumor progression, including tumor growth, distant metastasis, and chemoresistance development. Although there is a high correlation between Trp metabolism and tumor progression, it is unknown whether and how Trp metabolism affects the development of prostate cancer. In this study, we reported that the overexpression of Trp hydroxylase 1 (TPH1) caused the upregulation of Trp hydroxylation and mediated the production of 5-hydroxytryptamine (5-HT), contributing to tumor growth and poor prognosis in patients with prostate cancer. An increase in 5-HT levels triggered the activation of the Axin 1/β-catenin signaling pathway, thus enhancing cell proliferation and migration. Consequently, β-catenin cooperated with the Krüppel-type zinc finger family transcription factor ZBP-89 to upregulate TPH1 expression, further promoting Trp hydroxylation and forming the TPH1/5-HT/β-catenin/ZBP-89/THP1 positive feedback signaling loop. Interruption of the signaling loop by the THP1 inhibitor 4-chloro-dl-phenylalanine (PCPA) significantly improved anticancer effects and suppressed lung metastasis in prostate cancer–bearing mice. Our findings revealed a mechanism by which TPH1 promotes prostate cancer growth by inducing Trp hydroxylation and identified a novel THP1 target for an innovative prostate cancer therapeutic strategy.

## Introduction

Prostate cancer is the second most frequent malignant carcinoma in men, accounting for approximately 3.8% of all cancer-associated deaths (1). Persistent tumor growth and cancer metastasis are the major causes of complications and death in patients with prostate cancer (2, 3). Almost all patients who die of prostate cancer have distant metastatic (M) symptoms, including bone, lymph node, and lung metastasis (3). The prevention of distant metastasis and persistent tumor growth is an important goal in the clinical treatment of prostate cancer. However, the mechanism underlying prostate cancer metastasis and development remains poorly understood.

Cancer cells frequently display a high demand for nutrients and increased tryptophan (Trp) metabolism (4). Trp metabolism is tightly correlated with tumor progression in various cancer types. Because biological Trp metabolites participate in diverse physiological activities, such as nutrient synthesis, immune response, and activation of pro-survival signals, aberrant Trp metabolism has emerged as a primary cause of tumor progression (5). Trp is majorly catabolized by two signaling pathways, the indoleamine‐2, 3‐dioxygenase (IDO)/Trp‐2, 3‐dioxygenase (TDO)–dependent oxidative Trp/kynurenine signaling pathway and the TPH-dependent hydroxylation Trp–5‐hydroxytryptamine (5-HT) signaling pathway (6). According to reports, metabolites produced by oxidative Trp/kynurenine signaling pathways can promote the immunosuppression of the tumor microenvironment and mediate the activation of pro-survival signaling pathways in tumor cells, causing persistent tumor growth and metastasis in patients (7, 8). The oxidative Trp metabolic rate-limiting enzymes, IDO1 and TDO2, have been demonstrated to be constitutively expressed at high levels and are responsible for tumor growth and metastasis in several tumor types. More importantly, innovative therapeutic strategies targeting IDO1 have attracted increasing attention, in which IDO1 inhibitors showed remarkable results in tumor development suppression (9, 10). However, despite the conclusive evidence on IDO/TDO-induced tumor progression, limited information is available about the potential role of Trp hydroxylation in cancer development.

The hydroxylation of the Trp–5‐HT metabolic pathway is dependent on the rate‐limiting enzymes Trp hydroxylases (TPHs), including TPH1 and TPH2, which are capable of degrading Trp to 5-HT/serotonin (11). 5-HT is a neurotransmitter involved in a series of biological activities, including vasoconstriction, and central and peripheral actions. Based on findings, the expression of 5-HT may be correlated with the activation of pro-survival signaling pathways and tumor progression regulation. Sola-Penna et al. reported that 5-HT promoted glycolysis in human breast cancer cells and mediated the activation of the Jak1/STAT3/ERK1/2 signaling pathway (12). Furthermore, diverse studies have reported that 5−HT receptors expressed on tumor cells are closely associated with the development of breast cancer, suggesting that 5‐HT receptors may serve as a therapeutic target to improve tumor-suppressive effects (13). However, despite the advancement characterized by these findings, limited information is available about the role of the hydroxylation Trp–5‐HT metabolic pathway in tumor progression, and the mechanisms underlying the action of 5-HT and its receptors associated with tumor behavior remain unknown.

In our study, we initially confirmed the role of TPH1 in tumor development, in which TPH1 catabolized Trp to 5-HT and promoted prostate cancer proliferation/migration. Furthermore, we provided mechanistic evidence to suggest that 5-HT derived from Trp hydroxylation mediated the activation of the Axin/β-catenin signaling pathways. In turn, β-catenin cooperated with transcription factor zinc finger binding protein (ZBP)-89 to upregulate the expression of THP1, further promoting Trp hydroxylation and causing sustained tumor growth in prostate cancer. Interruption of the 5-HT/β-catenin/ZBP-89/TPH1 signaling loop by the 5-HT inhibitor significantly suppresses prostate cancer development. This therapeutic approach presented new insights for a better understanding of the role of THP1 and provided a novel strategy for reducing prostate cancer metastasis.

## Materials and methods

### Cell culture and reagents

Human prostate cancer cell lines DU145 and PC-3 were purchased from the American Type Culture Collection (ATCC, USA). TPH1-overexpressed DU145 and PC-3 cells were purchased from Cyagen Co. (China). All cells were maintained in a Roswell Park Memorial Institute 1640 complete culture medium (Gibco, USA) supplemented with 10% fetal bovine serum (FBS; Gibco, USA) in the presence of 5% CO_2_. Chemotherapeutic paclitaxel (PTX), TPH1 inhibitor 4-chloro-dl-phenylalanine (PCPA), and 5-HT were purchased from Sigma (USA). β-Catenin inhibitor LF3 was purchased from MedChemExpress (USA).

### Clinical specimens

The overall survival of patients with prostate cancer was obtained from https://www.cbioportal.org/. Paraffin sections of clinical prostate tumor tissues were obtained from the pathology laboratory of the Second Affiliated Hospital of Chongqing Medical University. Thirty patients with prostate cancer were divided into metastatic (M) and nonmetastatic (NM) groups according to the follow-up visit after surgical excision. Each patient provided written informed consent. All experiments were approved and monitored by the Ethical Committee of the Second Affiliated Hospital of Chongqing Medical University.

### Cell counting kit-8 assay

The proliferation of DU145 and PC-3 cells was assessed using a Cell Counting Kit-8 (CCK-8) assay kit (Solarbio, China) according to the manufacturer’s instructions. The mock or pretreated tumor cells were seeded in a 96-well plate at a density of 3,000 cells per well. CCK-8 solution was added and cell proliferation was examined at 0, 24, 48, and 72 h. Absorbance was quantified at 450 nm.

### Transwell assay

The cell migrating potential was determined using a transwell assay. Briefly, 10^5^ DU145 and PC-3 cells were added into an 8-μm transwell chamber with 500 μl of a culture medium supplemented with 10% FBS. To each well, 1 ml of a culture medium was added. After a culture of 24 h, the chamber was fixed with ice ethanol and stained with crystal violet solution for 30 min. The migrating cells were counted under the microscope.

### RNA interference

ZBP-89 was silenced in DU145 and PC-3 using small interfering RNA (siRNA). The siRNAs targeting ZBP‐89 (siRNA1: 5′‐AAGATCGAAGTATGCCTCACCTT‐3′ and siRNA2: 5′‐AAGATCGAACGTGTCCTCACCTT‐3′) were purchased from Ruibo Co. (China). Silencing of ZBP-89 was performed according to the manufacturer’s instructions. The efficiency of ZBP-89 silencing was examined using quantitative polymerase chain reaction (qPCR). The primers targeting ZBP-89 were as follows: forward primer 5′-CAGGACAATGGTTGTAATGGGT-3′ and reverse primer 5′-GGTGAGGCATACTTCGATCTTGA-3′. Glyceraldehyde 3-phosphate dehydrogenase was used for normalization.

### Western blotting

Cellular proteins were extracted using the Nonidet P-40 lysis buffer (Thermo Fisher Scientific, USA) supplemented with a proteinase inhibitor (Thermo Fisher Scientific, USA). Twenty micrograms of proteins was separated by 10% sodium dodecyl sulfate–polyacrylamide gel electrophoresis and visualized with an enhanced luminol-based chemiluminescent kit (Millipore, USA). The primary antibodies used were those against human THP1 (1:1,000; Abcam, UK), Axin 1 (1:1,000; Abcam, UK), and β-catenin (11:1,000; Abcam, UK). β-Actin (1:2,000; Abcam, UK) was used as the protein loading control.

### Immunofluorescence

The dewaxed specimens were blocked with Tris-buffered saline supplemented with 5% bovine serum albumin for 30 min and were then treated overnight with primary antibodies: anti-TPH1 (1:200; Abcam, UK) and anti–β-catenin (1:200; Abcam, UK) at 4℃. A fluorescent secondary antibody (Thermo, USA) was used for treating the sample for 2 h at room temperature. Fluorescence images were visualized under a confocal microscope (Leica, Germany). The intensity of protein expression was determined using ImageJ 2.0.

### Enzyme-linked immunosorbent assay

The concentrations of 5-HT and Trp in the culture medium were measured using an enzyme-linked immunosorbent assay (ELISA). A human 5-HT ELISA kit was purchased from CUSABIO Co. (China). A human Trp ELISA kit was purchased from Biocompare (USA). For 48 h, 10^5^ DU145/PC-3 cells were cultured in 2 ml of the culture medium. The culture medium for 0/48 h was collected for 5-HT/Trp concentration quantification according to the manufacturer’s instructions.

### Animal experiment protocols

Nonobese diabetic/severe combined immunodeficiency (NOD-SCID) mice aged 6–8 weeks were purchased from Huafukang (China) and maintained in a specific pathogen-free facility. For the subcutaneous prostate cancer model, 10^6^ TPH1-overexpressing PC-3 cells were subcutaneously injected into NOD-SCID mice. When the tumor had grown to 500 mm^3^, the mice were treated with phosphate-buffered saline (PBS), PCPA (5 mg/kg), PTX (10 mg/kg), and a combination every 4 days. The tumor volume and overall survival of tumor-bearing mice were recorded every day (n = 6 in each group). Tumor volume = length × width^2^/2. Some mice were sacrificed on day 20 for lung metastasis analysis (n = 6 per group). All animal experiments were performed and monitored per the guidelines approved by the Animal Care and Use Committee of the Second Affiliated Hospital of Chongqing Medical University.

### Statistical analysis

Each experiment was repeated three times independently. Results are presented as means ± SEM. Differences between the two groups were determined using a *t*-test with GraphPad 6.0. The survival rates were determined by Kaplan–Meier survival analysis (*p < 0.05; **p < 0.01; ***p < 0.001; ns, no significant difference).

## Results

### TPH1 drives prostate cancer cell proliferation and migration

**Figure 1:**
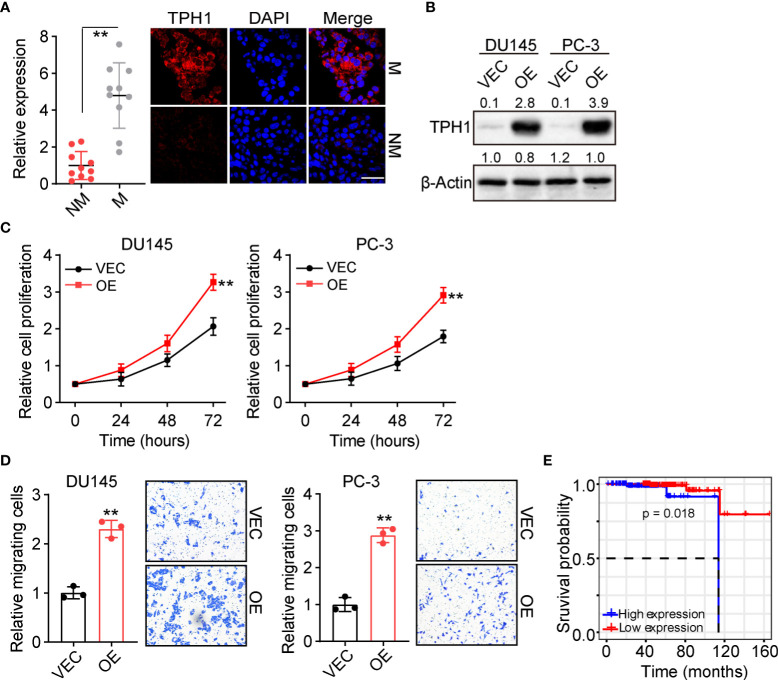
Tryptophan hydroxylase 1 (TPH1) drives prostate cancer cell proliferation and migration. **(A)** Immunofluorescence staining of TPH1 in tumor tissues from patients with metastatic (M) and nonmetastatic (NM) prostate cancer (n = 10 per group). The scale bar is 60 μm. **(B)** Western blotting of TPH1 in DU145/PC-3 and TPH1-overexpressing DU145/PC-3 cells. **(C)** Relative proliferation of DU145/PC-3 and TPH1-overexpressing DU145/PC-3 cells using the CCK-8 assay. **(D)** Relative number of migrating DU145/PC-3 and TPH1-overexpressing DU145/PC-3 cells determined using the transwell assay. **(E)** Overall survival of patients with prostate cancer divided into the high TPH1 (n = 96) and low TPH1 expression (n=150) groups. **p < 0.01.

As reported previously, tumor cells with enhanced proliferative properties usually exhibit an aberrant Trp metabolic signature and distinct expression of metabolic enzymes. To assess the level of Trp metabolism, or more specifically, the role of TPH1 in the development of prostate cancer, we examined the expression of TPH1 in prostate tumor tissues. Patients with prostate cancer were divided into M and NM groups according to the follow-up visit following surgical excision. The orthotopic tumor tissues were collected and the expression of TPH1 was assessed using immunofluorescence. Intriguingly, tumor cells in the M group showed elevated expression of TPH1 when compared to those in the NM group (**Figure 1A**). The prostate cancer cells DU145 and PC-3 exhibit stronger proliferative characteristics when TPH1 was overexpressed (**Figures 1B, C**). This indicated that TPH1 may promote the proliferation of prostate cancer cells to induce tumor progression. Transwell analysis was performed to examine the migration of DU145/PC-3 cells with or without TPH1 overexpression to better understand the role of TPH1 in cell migration. As anticipated, TPH1 overexpression significantly promoted the migration of DU145 and PC-3 cells (**Figure 1D**), indicating that TPH1 promoted the potential for cell migration in prostate cancer. This result is consistent with that of M tumor tissues in **Figure 1A**. Furthermore, we sought to assess the influence of TPH1 on the overall survival of patients with prostate cancer. Patient information was collected from The Cancer Genome Atlas database, and the overall survival of patients was evaluated according to the expression of TPH1. Interestingly, a negative correlation was observed between the survival time and TPH1 expression in patients with prostate cancer (**Figure 1E**). These results suggested that TPH1 promotes the proliferation and migration of prostate cancer cells, causing a poor prognosis in prostate cancer.

### TPH1 promotes Trp hydroxylation and 5-HT production

**Figure 2:**
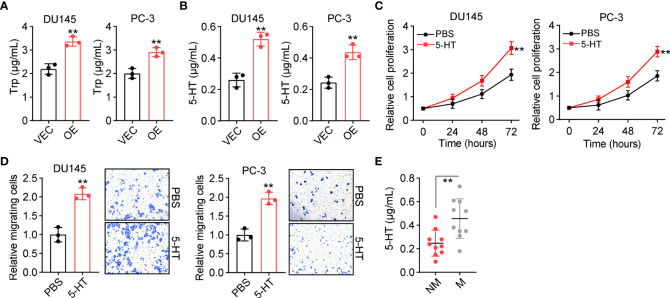
TPH1 promotes tryptophan (Trp) hydroxylation and 5-hydroxytryptamine (5-HT) production. **(A)** Culture of 10^5^ DU145/PC-3 and TPH1-overexpressing DU145/PC-3 cells in 2 ml of culture medium for 48 h. Trp consumption was examined using a human Trp enzyme-linked immunosorbent assay (ELISA) kit. **(B)** 5-HT production in **(A)** was examined using a human 5-HT ELISA kit. **(C)** Relative proliferation of DU145/PC-3 cells treated with phosphate-buffered saline (PBS) or 5-HT (5 μM). **(D)** Relative number of migrating DU145/PC-3 cells treated with PBS or 5-HT (5 μM). **(E)** Quantification of 5-HT in tumor tissues from patients with M and NM prostate cancer (n = 10 per group) using ELISA. **p < 0.01.

We further explored the underlying mechanism of TPH1-induced prostate cancer progression. TPH1 belongs to the family of aromatic amino acid hydroxylases, which can catalyze the hydroxylation of L-Trp to 5-HT (14). Intriguingly, an increasing number of studies have reported that 5-HT expression is closely correlated with cell proliferation in hepatic carcinoma (15). This prompted us to consider that TPH1 may mediate Trp hydroxylation and produce 5-HT to stimulate prostate cancer development. Therefore, we cultured 10^5^ DU145/PC-3 and TPH1-overexpressing DU145/PC-3 cells in 2 ml of the culture medium for 48 h and subsequently examined the Trp consumption and 5-HT production in the culture medium. Consistent with our hypothesis, Trp metabolism and 5-HT levels were higher in TPH1-overexpressing DU145/PC-3 cells than in the vector groups (**Figures 2A, B**), suggesting that TPH1 facilitated Trp hydroxylation in prostate cancer cells. To further assess the role of 5-HT in prostate cancer development, 5-HT was added into the culture medium of DU145 and PC-3 cells, and cell proliferation and migration were assessed. Both DU145 and PC-3 cells displayed enhanced proliferative (**Figure 2C**) and migrative (**Figure 2D**) properties following 5-HT treatment. The results indicate that 5-HT contributed to tumor progression induced by TPH1. Accordingly, we further detected the secretion of 5-HT in the tumor tissues of patients and observed that patients with M prostate cancer had higher 5-HT expression than patients in the NM group (**Figure 2E**). Collectively, the results suggested that 5-HT derived from Trp hydroxylation caused prostate cancer development.

### 5-HT mediates axin/β-catenin activation in prostate cancer

**Figure 3:**
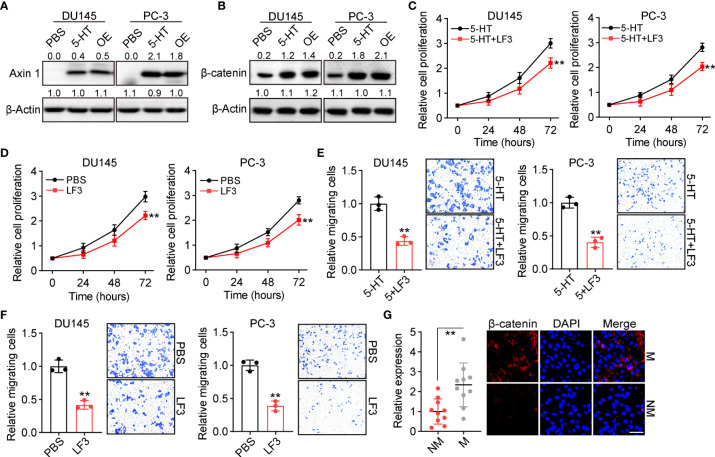
5-HT mediates Axin/β-catenin activation in prostate cancer. **(A)** Western blotting of Axin 1 in DU145/PC-3 cells (treated with PBS or 5 μM 5-HT) and TPH1-overexpressing DU145/PC-3 cells. **(B)** Western blotting of β-catenin in DU145/PC-3 cells (treated with PBS or 5 μM 5-HT) and TPH1-overexpressing DU145/PC-3 cells. **(C)** Proliferation of DU145/PC-3 cells treated with 5-HT (5 μM) combined with LF3 (2 μM) or not. **(D)** Proliferation of TPH1-overexpressing DU145/PC-3 cells treated with PBS or LF3 (2 μM). **(E)** Relative number of migrating DU145/PC-3 cells treated with 5-HT (5 μM) combined with LF3 (2 μM) or not. **(F)** Relative number of migrating TPH1-overexpressing DU145/PC-3 cells treated with PBS or LF3 (2 μM). **(G)** Immunofluorescence staining of β-catenin in tumor tissues from patients with M and NM prostate cancer (n = 10 per group). The scale bar is 60 μm. **p < 0.01.

Given the essential role of 5-HT in prostate cancer development, we sought to understand how 5-HT controls tumor progression *in vivo*. The 5-HT–associated receptor has previously been reported to promote colon cancer metastasis through β-catenin signaling (16). In this study, we aimed to evaluate whether β-catenin signaling contributes to tumor progression induced by 5-HT. We examined the expression of Axin and β-catenin in 5-HT–treated or TPH1-overexpressing DU145/PC-3 cells. Intriguingly, both 5-HT treatment and TPH1 overexpression increased the expression of Axin (**Figure 3A**) and β-catenin (**Figure 3B**), indicating that TPH1-associated Trp hydroxylation promoted the activation of β-catenin. Subsequently, to further investigate the pro-tumor effects of β-catenin signaling, the β-catenin inhibitor LF3 (17) was applied to treat tumor cells (5-HT pretreatment or TPH1 overexpression), and cell proliferation/migration was detected. Consistent with our hypothesis, the inhibition of β-catenin suppressed cell proliferation in 5-HT–treated DU145/PC-3 cells (**Figure 3C**) or TPH1-overexpressing DU145/PC-3 cells (**Figure 3D**). Similar results were observed in the migration assay (**Figures 3E, F**), confirming that 5-HT controlled prostate cancer development *via* β-catenin signaling. Collectively, greater β-catenin expression was observed in the M tissues from patients with colorectal cancer than in the NM group (**Figure 3G**). The results indicated that 5-HT–mediated β-catenin activation promotes prostate cancer progression.

### β-catenin cooperates with ZBP-89 to promote TPH1 and forms a signaling feedback loop

**Figure 4:**
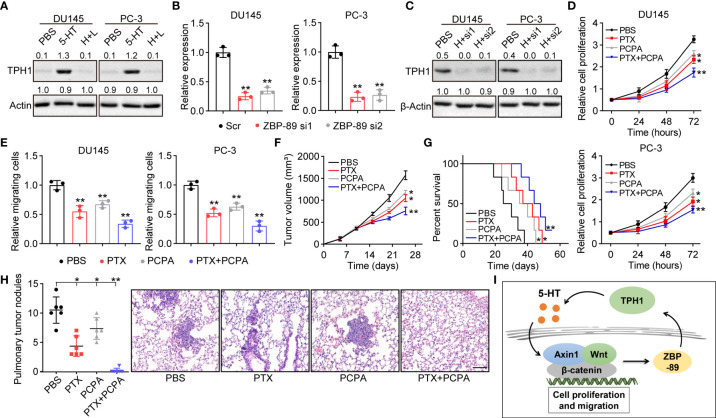
β-Catenin cooperates with zinc finger binding protein (ZBP)-89 to promote TPH1 and forms a signaling feedback loop. **(A)** Western blotting of TPH1 in DU145/PC-3 cells treated with PBS, 5-HT (5 μM), and 5-HT (5 μM) combined with LF3 (2 μM). **(B)** Relative expression of ZBP-89 in DU145/PC-3 cells treated with Scr and ZBP-89 siRNA, as determined by quantitative polymerase chain reaction (qPCR). **(C)** Western blotting of TPH1 in DU145/PC-3 cells treated with 5-HT (5 μM) and 5-HT (5 μM) combined with ZBP-89 siRNA. **(D)** Proliferation of TPH1-overexpressing DU145/PC-3 cells treated with PBS, paclitaxel (PTX) (0.5 μg/ml), 4-chloro-dl-phenylalanine (PCPA) (2 μM), and a combination of the same. **(E)** Relative number of migrating TPH1-overexpressing DU145/PC-3 cells treated with PBS, PTX (0.5 μg/ml), PCPA (2 μM), and a combination of the same. **(F)** Tumor volume of TPH1-overexpressing PC-3–bearing mice treated with PBS, PTX, PCPA, and a combination of the same. **(G)** Overall survival of TPH1-overexpressing PC-3–bearing mice treated with PBS, PTX, PCPA, and a combination of the same. **(H)** M nodule numbers and hematoxylin and eosin (H&E) staining of lung tissues from TPH1-overexpressing PC-3–bearing mice treated with PBS, PTX, PCPA, and a combination of the same. The scale bar is 100 μm. **(I)** Schematic diagram of the 5-HT/β-catenin/ZBP-89/TPH1 signaling loop in prostate cancer. *p < 0.05; **p < 0.01.

ZBP-89, a Krüppel-type zinc finger family transcription factor, has been demonstrated to cooperate with Wnt β-catenin signaling to promote the expression of TPH1 in the inflammatory response of mice (18). This indicated the potential presence of a 5-HT/β-catenin/ZBP-89/TPH1 signaling feedback loop in prostate cancer. To confirm our hypothesis, TPH1 expression was determined by Western blotting in 5-HT–treated DU145/PC-3 cells. Intriguingly, TPH1 was upregulated in the 5-HT–treated group; however, the upregulation of TPH1 was reversed by the β-catenin inhibitor LF3 (**Figure 4A**). Meanwhile, to determine the role of ZBP-89 in TPH1 regulation, a siRNA was added to silence ZBP-89 in DU145 and PC-3 cells (**Figure 4B**). Similarly, ZBP-89 inhibition caused the downregulation of TPH1 in 5-HT–treated cells (**Figure 4C**), indicating that β-catenin and ZBP-89 cooperated to mediate the upregulation of TPH1, thus forming a positive feedback loop to drive prostate cancer development.

Given the crucial role of the 5-HT/β-catenin/ZBP-89/TPH1 signaling loop in prostate cancer development, interrupting the TPH1 signaling loop for improving the tumor-suppressive effects in TPH1 overexpression in prostate cancer may be possible. In this study, we combined chemotherapeutic PTX with PCPA, a THP1 inhibitor, to examine anticancer effects. PCPA significantly suppressed the proliferation and migration of DU145 and PC-3 cells. Meanwhile, inhibition of THP1 improved the tumor-suppressive effects of PTX (**Figures 4D, E**), indicating the synergistic effect of PCPA and PTX in prostate cancer treatment. To further confirm the anticancer effects *in vivo*, a subcutaneous prostate tumor-bearing mice model was established by subcutaneously injecting 10^6^ TPH1-overexpressing PC-3 cells into immunodeficient mice. When the tumor had grown to 500 mm^3^, the mice were treated with PBS, PCPA, PTX, and PCPA combined with PTX. The combined therapeutic strategy exhibited a stronger tumor-suppressive effect that inhibited tumor growth (**Figure 4F**) and prolonged the survival (**Figure 4G**) of tumor-bearing mice. Considering the migrative properties of THP1-overexpressing prostate cancer cells, we further examined lung metastasis of PC-3 cells in a different group. Both PTX and PCPA treatment reduced the M lung nodules in tumor-bearing mice. However, the combined group showed a superior inhibitory effect, in which no M nodules were observed in PCPA+PTX-treated mice (**Figure 4H**). Collectively, the results suggested that the interruption of the 5-HT/β-catenin/ZBP-89/TPH1 signaling loop by the THP1 inhibitor PCPA efficiently improved the outcome of prostate cancer therapy; this finding constitutes a novel perspective in tumor treatment.

## Discussion

Tumor cells frequently exhibit a peculiar metabolic status, and metabolic phenotypes of cells with tumor tissues are heterogeneous. In most situations, tumor cells catabolize glucose at much higher rates than normal cells, indicating an increased demand for nutrients, including Trp and its metabolites (19). The enhanced metabolism of Trp has been demonstrated to be closely associated with poor prognosis in patients with cancer (5). However, previous studies have majorly focused on the oxidative Trp/kynurenine signaling pathway and confirmed the crucial role of IDO/TDO in regulating immunosuppression and tumor progression (20–22). In this study, we further identified the role of TPH-dependent hydroxylation in the Trp–5‐HT signaling pathway in prostate cancer development. We provided evidence that the overexpression of THP1 enzymes can promote Trp hydroxylation and 5-HT production, thereby enhancing the proliferation and migration of prostate cancer cells. This investigation was conducted to further elucidate the TPH metabolic signaling pathway involved in the process of prostate tumor progression.

It has been established that the presence of 5-HT in the tumor microenvironment is essential for tumor growth and potentially associated with the migration of cancer cells (23, 24). Meanwhile, the activation of 5-HT receptors, such as 5-HT1 and three receptors, occurs preferentially in tumor cells and can promote cancer progression in several tumor types (8, 25). Diverse pro-survival signaling pathways have been reported to undergo increased activation in response to 5-HT and its receptors (16, 26). For example, Fatima and colleagues reported that 5-HT can promote hepatocellular carcinoma growth by influencing the β-catenin signaling pathway (27). In addition, Banes et al. demonstrated that 5-HT mediates the activation of the JAK/STAT pathway in vascular smooth muscles (28). Consistent with these findings, our study showed the activation of the Axin 1/β-catenin signaling pathway in TPH1 overexpression and 5-HT–treated prostate cancer cells. Furthermore, Trp metabolism and 5-HT levels were presumably elevated in TPH1-overexpressing DU145/PC-3 cells, which caused the sustained proliferation of prostate cancer cells. Intriguingly, the upregulation of β-catenin signaling induced by TPH1/5-HT also promoted cell migration and metastasis in prostate cancer. Previous findings have confirmed that 5-HT correlates with the migration of vascular endothelial cells and plays a vital role in wound repair (29). However, information about the roles of 5-HT in cancer metastasis is limited. Our study provided further evidence to suggest the role of 5-HT in cell migration regulation and elucidated the mechanism underlying prostate cancer metastasis, which depended on the Axin/β-catenin signaling pathway.

The activation of β-catenin signals is critical in diverse tumor types (30), and we further observed that β-catenin mediates the upregulation of TPH1 in prostate cancer, forming the 5-HT/β-catenin/TPH1 signaling loop. The Krüppel-type zinc finger family transcription factor ZBP-89 regulates TPH1 expression and mediates the defense against *Salmonella typhimurium* in mice (18, 31). In our study, we observed that 5-HT can further upregulate the expression of TPH1 through β-catenin signaling; however, both ZBP-89 and β-catenin inhibition suppressed the upregulation of TPH1 in DU145 and PC-3 cells. This indicated that β-catenin cooperated with ZBP-89 to promote TPH1 expression, which in turn facilitated Trp hydroxylation and helped establish the TPH1/5-HT/β-catenin/TPH1 metabolic signaling loop. Therefore, we sought to suppress TPH1 to interrupt the 5-HT/β-catenin/TPH1 signaling loop to suppress tumor growth and eliminate M tumor cells. In our study, the TPH1 inhibitor PCPA was combined with the chemotherapeutic agent PTX, which revealed improved tumor-suppressive effects *in vitro* and *in vivo*. More importantly, PCPA efficiently suppressed cell migration and distant lung cell metastasis in PC-3–bearing mice. Our results were consistent with previous clinical and experimental data. Asada and colleagues reported that the depletion of 5-HT and selective inhibition of its receptor can suppress angiogenesis and tumor initiation in Lewis lung cancer (31). Additionally, Christensen et al. reported that selective 5-HT reuptake inhibitors can inhibit the activation of Ki-67 and improve the outcomes in epithelial ovarian cancer (32). These results strengthened our concept that the blockade of the TPH1/5-HT axis suppresses tumor development.

In conclusion, our findings demonstrated that Trp hydroxylation induced by TPH1 plays an essential role in prostate cancer development, which was dependent on a 5-HT/β-catenin/ZBP-89/TPH1 signaling loop (**Figure 4I**). The targeting of TPH1 led to a stronger anticancer effect and suppression of distant metastasis, providing innovative insights for prostate cancer therapy.

## Data availability statement

The original contributions presented in the study are included in the article/Supplementary Material. Further inquiries can be directed to the corresponding author.

## Ethics statement

This study was reviewed and approved by Ethical Committee of the Second Affiliated Hospital of Chongqing Medical University. The patients/participants provided their written informed consent to participate in this study. The animal study was reviewed and approved by the Animal Care and Use Committee of the Second Affiliated Hospital of Chongqing Medical University.

## Author contributions

CG and GX contributed to conception and design of the study. CG and JY carried out the experiment. XY performed the data analysis. GX reviewed the article. All authors contributed to the article and approved the submitted version.

## Conflict of interest

The authors declare that the research was conducted in the absence of any commercial or financial relationships that could be construed as a potential conflict of interest.

## Publisher’s note

All claims expressed in this article are solely those of the authors and do not necessarily represent those of their affiliated organizations, or those of the publisher, the editors and the reviewers. Any product that may be evaluated in this article, or claim that may be made by its manufacturer, is not guaranteed or endorsed by the publisher.
